# Improvement of Cell Growth of Uterosacral Ligament Fibroblast Derived from Pelvic Organ Prolapse Patients by Cold Atmospheric Plasma Treated Liquid

**DOI:** 10.3390/cells10102728

**Published:** 2021-10-13

**Authors:** Ihn Han, Seung Ah Choi, Seul I Kim, Eun Ha Choi, Young Joo Lee, Youngsun Kim

**Affiliations:** 1 Plasma Bioscience Research Center, Applied Plasma Medicine Center, Kwangwoon University, Seoul 01897, Korea; hanihn@kw.ac.kr (I.H.); ehchoi@kw.ac.kr (E.H.C.); 2 Biomedical Research Institute, Seoul National University Hospital, Seoul 03080, Korea; aiipo@snu.ac.kr; 3 SeoulinMedicare Company, Hwaseong-si 18469, Korea; sulee88@naver.com; 4 Department of Obstetrics and Gynecology, Kyung Hee University Medical Center, Seoul 02447, Korea; intro4med@naver.com

**Keywords:** pelvic organ prolapse, human uterosacral ligament fibroblasts, non-thermal biocompatible plasma, plasma-treated liquid, p53 apoptotic signal

## Abstract

Pelvic organ prolapse (POP) is a chronic disorder that affects quality of life in women. Several POP treatments may be accompanied by abrasion, constant infection, and severe pain. Therefore, new treatment methods and improvements in current treatments for POP are required. Non-thermal atmospheric-pressure plasma is a rising biomedical therapy that generates a mixed cocktail of reactive species by different mechanisms. In this study, we applied a cylinder-type dielectric barrier discharge plasma device to create a plasma-treated liquid (PTL). The PTL was added to primary cultured human uterosacral ligament fibroblast (hUSLF) cells from POP patients at each stage. Surprisingly, treatment with diluted PTL increased hUSLF cell viability but decreased ovarian cancer cell viability. PTL also decreased cell apoptosis in hUSLF cells but increased it in SKOV3 cells. Our results suggest that PTL protects hUSLF cells from cell apoptosis by controlling the p53 pathway and improves cell viability, implying that PTL is a promising application for POP therapy.

## 1. Introduction

The pelvic connective tissue, which supports the pelvic organs, includes the round ligament and the uterosacral ligament. The latter are key structures in the pelvic support system and establish level 1 support of the cervix and upper vagina, while the former is attached high in the pelvis to the fundus of the uterus and less associated with the pelvic support system [1]. In vitro studies reported that the cervical portions of the uterosacral ligament support more than 17 kg of weight before failing [2]. A universal health problem, pelvic organ prolapse (POP) is among the most frequent gynecological chronic disorders. POP is defined as “the genesis of single or additional of the anterior wall of vagina, posterior wall of vaginal, the cervix or the peak of the vagina” [3]. A loss of regular connection and support leads to entry of the pelvic organs into the vaginal canal [4], affecting 50% of all women over 50 years of age [5]. Rarely, the pelvic organs directly cause life-threatening disease due to weakening of the pelvic support structure (muscle, fascia, and tongue), causing the uterus, bladder, rectum, and small intestine to protrude from the vagina [6,7,8,9,10]. However, the majority of women with POP are asymptomatic. Symptoms typically include the urinary, vaginal, or bowel organs. The composition of the female pelvic floor was thoroughly described previously [11,12]. However, POP can lead to physical impairments such as chronic pain, a heavy feeling in the pelvis, urinary incontinence, bowel incontinence, and sexual dysfunction, which can limit a woman’s activities and affect her quality of life. The risk of a woman developing POP increases by 3.2 times when her mother is affected and 2.4 times when her sister is affected [13]. POP is the most common indication for gynecological surgery in postmenopausal women. However, its failure rate is comparatively high: an expected 30% of women require reoperation [14]. Different varieties of vaginal implants, absorbable and non-absorbable, have been introduced in pelvic floor reconstructive surgeries but have many severe adverse effects. Until recently, surgical reconstruction using non-degradable, lightweight mesh slings was the most common treatment option for POP [15]; however, due to unacceptable post-surgical complications, these products have since been banned in Australia, New Zealand, the United States, and the United Kingdom [16]. Current news has highlighted a critical need for new strategies that can ensure secure and successful therapy for women with POP.

Non-thermal atmospheric-pressure plasma has been investigated for numerous therapeutic applications [17]; so far, it can be used directly on existing tissues and cells [18], death of bacteria, and induction of coagulation of blood without significant heating [19,20,21]. Non-thermal atmospheric-pressure plasma is composed of components, including neutrons, electrons, ions, cations, molecules, radicals, light, and electromagnetic effects as the fourth matter state. Ions and radicals in the plasma are highly reactive with other molecules and atoms, so they easily penetrate the surface of the material and react with materials that contribute to surface modification. Plasma applications in biology and medicine use non-thermal biocompatible plasma (NBP) sources, which are suitable for biological targets such as food, cosmetics, and biomaterials and improve human health and quality of life, including the characteristics of the interaction between plasma and inorganic and organic substances. This is a new field in which the basic source characteristics of interaction are clarified, and the multidisciplinary basics for the medical application of plasma are actively pursued. NBP is in the spotlight as a new technology in therapeutic fields such as cancer treatment, the suppression of skin infections, the promotion of tissue regeneration, and immune regulation. Plasma-treated liquid/solution (PTL/PTS), so-called plasma activated liquid/plasma activated solution (PAL, PAS), often also known as plasma activated water (PAW), is created by adding plasma to liquids such as water, phosphate-buffered saline (PBS), lactated Ringer’s solution, and cell culture medium. PTL has the enormous advantage of being easier to apply to tissues and organs than existing treatment devices. However, the PTL-treated standard for each disease has not yet been established, and our understanding of its mechanism is insufficient.

To address this issue, we must develop a treatment protocol and database for PTL effectiveness as a new therapeutic application that might suppress the apoptosis of cells derived from POP patients due to NBP. In this study, we used a cylinder-type dielectric barrier discharge (c-DBD) plasma source developed by our laboratory for agricultural and food applications [22].

## 2. Materials and Methods

### 2.1. Biopsy Collection of POP Tissues

Human uterosacral ligament tissue samples (n = 40) were collected from patients after each provided informed consent with approval from the Ethics Committee of Kyung Hee University Hospital, Seoul, Korea (no. 2020-02-028). Full-thickness biopsy specimens from the anterior vaginal wall were obtained from women with POP during laparoscopic-assisted vaginal hysterectomy. According to a POP quantitative examination [23], tissue samples of patients with POP stages 2 and 3 (n = 20) who underwent hysterectomy as part of pelvic reconstruction surgery were included in the POP group. The control group comprised 20 patients who underwent hysterectomy for cervical intraepithelial neoplasia or dysfunctional uterine bleeding. There were no differences in basic characteristics between the two groups (Table 1). The tissues were immediately collected and washed with cold PBS. All tissue samples were prepared within 6 h.

### 2.2. Human Uterosacral Ligament Fibroblast Primary Cell Cultures

Human uterosacral ligament fibroblasts (hUSLF) were isolated from POP tissues after hysterectomy. The tissues were placed in a 100 mm culture dish and chopped with 10 and 15 blades. The cells were isolated after being digested with type I collagenase and dispase using the primary culture method established by I. Han [24]. Primary cultured hUSLF cells were cultured in alpha Modified Eagle’s Minimum Essential Media (α-MEM; HyClone, South Logan, UT, USA) supplemented with 10% fetal bovine serum (FBS; Gibco, Thermo Fisher, San Jose, CA, USA), 1% penicillin/streptomycin (100 U/mL penicillin and 100 µg/mL streptomycin; Sigma-Aldrich, St. Louis, MO, USA), 1% fungizone (Gibco, Thermo Fisher, San Jose, CA, USA), and 10 ng/mL basic fibroblast growth factor (Corning Costar, NY, USA). SKOV3 and OVCA429 cancer cells were cultured in Dulbecco’s modified Eagle’s medium (DMEM; Welgene, Seoul, Korea) supplemented with 1 g/L glucose, 10% FBS (Gibco), and 1% antibiotics (Gibco). The primary hUSLF cells were used within passage 10 for all experiments.

### 2.3. Production of PTL

**Figure 1:**
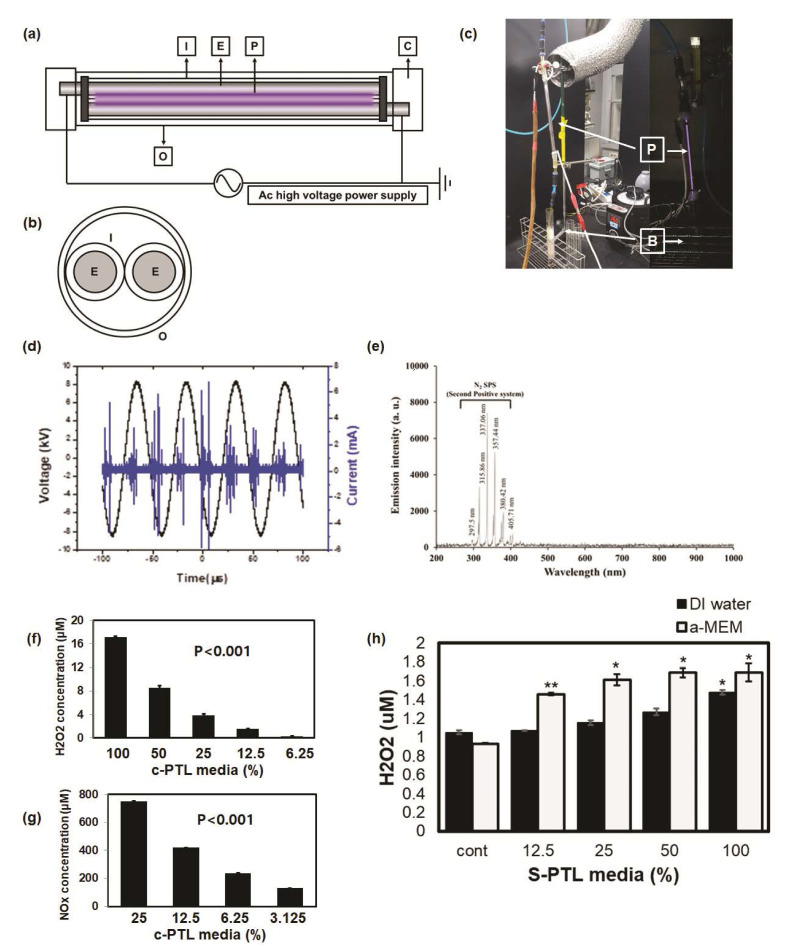
Characterization of plasma device for plasma-treated liquid (PTL). Front view (**a**) and cross-sectional view (**b**) of cylinder-type dielectric barrier discharge plasma device (**c**). I, inlet quartz tube; E, electrode; P, plasma discharged; C, connector with bubbler system; O, outlet quartz tube; B, bubbler system. Current and voltage waveforms (**d**) of the plasma discharge and the spectrum (**e**) of the plasma driven 8 kV p-p at 20 kHz with air gas flow rate is 1lpm. Hydrogen peroxide (**f**) and nitric oxide (**g**) were measured according to the dilution percentage from 50% to 3.125% (PTL/serum-free cell culture medium). For comparison, the hydrogen peroxide concen-trations were measured in the deionized water and cell culture medium (**h**). The level of signifi-cance was denoted as follows: * *p* < 0.05; ** *p* < 0.01.

Figure 1 shows a schematic image of a c-DBD connected to an underwater bubbler system [22]. Figure 1a shows that the structure of c-DBD consists of two brass electrodes surrounded by an inlet and outlet quartz tube connected to the connector and bubble diffuser. The plasma discharge was generated by connecting two brass electrodes divided by a quartz tube (Figure 1b). Air gas (1 L/min) was supplied to the plasma device through the air gas line using 150 V of power (Figure 1c). For plasma generation, electric power was provided by an alternating current (AC) power supply (20 kHz). The c-DBD plasma was exposed for 20 min to α-MEM and DMEM without serum, FBS. A total of 20 mL of PTL was treated. The PTL was freshly produced before the start of each experiment.

### 2.4. Measurement of Reactive Species of PTL

Extracellular levels of H_2_O_2_ and NOx were measured by using the QuantiChrom TM Peroxide assay kit and Nitric oxide assay kit (BioAssay systems, Hayward, CA, USA), respectively, within the cell culture media. Serum free α− MEM medium 119 was evaluated after giving NBP treatment using air, and the reactive species were detected after 20 min of treatment time. The experiment procedures were performed following the kit instructions, and the absorbance of H_2_O_2_ was calculated at 585 nm and 540 nm for NOx. Components of cell culture media are listed in Supplementary Table S1.

### 2.5. Cell Viability Assay

When the primary cultured hUSLF cells reached 80~90% confluence in the culture dishes, adherent cells were detached using 0.25% trypsin-ethylenediaminetetraacetic acid (Welgene) and then passaged or seeded. The cells were seeded at a density of 5000 cells/well in 96-well plates and incubated for 24 h. The PTL was treated at different diluted doses of PTL: culture media of 1:1, 1:2, 1:4, 1:8, and 1:16. The cell viability assay was performed using EZ-Cytox (DoGenBio, Seoul, Korea) according to the manufacturer’s protocol. OD was measured using a Synergy HT Microplate Reader (Bio-Tek Instruments Inc., Winooski, VT, USA) at a wavelength of 450 nm. Cell viability was calculated as (treatment/control group) × 100%.

### 2.6. Apoptosis Assay

To determine apoptosis, cells were seeded at a density of 2 × 10^5^ cells/well in 6-well plates. Briefly, seeded cells were treated with PTL; after 24 h of incubation, the cells were collected by washing twice with cold PBS and stained with Annexin V-FITC and propidium iodide. Cell apoptosis was measured using a FITC Annexin V Apoptosis Detection Kit II (BD Pharmingen, San Diego, CA, USA) according to the manufacturer’s instructions (BD Biosciences, San Jose, CA, USA) and analyzed using flow cytometry. Apoptotic cells were detected using a FACSVerse instrument (BD Biosciences).

### 2.7. Immunoblotting

The hUSLF cells were seeded in 6-well plates after incubating for 24 h, and then reacted with various ratios of PTL mixed with DMEM for 24 h in a CO_2_ cell culture incubator. The cells were harvested, washed with cold PBS, and lysed with RIPA buffer (GenDEPOT, Barker, TX, USA) containing a cocktail of protease and phosphatase inhibitors (Sigma, St. Louis, MO, USA). After protein quantification using a modified Lowry assay kit (DC protein assay, Bio-Rad Laboratories, Hercules, CA, USA), the protein samples were loaded for sodium dodecyl sulfate–polyacrylamide gel electrophoresis. For the Western blot assay, PARP, p53, Bax, Cdc2, and GAPDH proteins were identified using rabbit monoclonal PARP antibody (1:1000 dilution; Cell Signaling Technology, Inc., Denver, MA, USA), rabbit monoclonal p53 antibody (1:1000 dilution; Cell Signaling Technology, Inc., Denver, MA, USA), rabbit monoclonal Bax antibody (1:1000 dilution; Bio-rad, Hercules, CA, USA), mouse monoclonal Cdc2 antibody (1:1000 dilution; Bio-Rad), and rabbit polyclonal GAPDH antibody (1:1000 dilution; Bio-Rad) as primary antibodies, respectively, in combination with anti-mouse or rabbit secondary antibody conjugated with horseradish peroxides (1:2000 dilution; Cell Signaling Technology, Inc., Denver, MA, USA). The membranes were developed using Clarity Western ECL substrate (Bio-Rad) according to the manufacturer’s instructions. Images were obtained with ChemiDocTM Touch Gel Imaging System (Bio-Rad).

### 2.8. Statistical Analysis

All data were obtained from at least three independent experiments. Values from the experiments are presented as mean ± standard deviation. The data were analyzed using Prism software. Differences were considered significant at *p* ≤  0.05.

## 3. Results

### 3.1. Plasma Device for PTL

We used c-DBD to create the PTL as shown in Figure 1a. The atmospheric-pressure plasma device used to create the PTL is composed of a metal electrode outside (O), such as a brass piece in the form surrounding the internal fluid tube (quartz tube, I) that causes a plasma discharge between the electrodes connected to the high-voltage AC power source. As shown in Figure 1b, the electrode pair is surrounded once more by the external dielectric tube (E), and the injected discharge base is configured to pass only the plasma generation period. The gas supplied is ionized by the plasma to produce free radical species and activated species and is injected into the resulting active species in the medium to create a PTL that is used as a diluted plasma solution. The control voltage is driven by an AC power supply reaching 150 V at a frequency of 20 kHz. The electrode diameter is 20 mm, the diameter of the internal dielectric tube is 20 mm, the diameter of the outer dielectric tube is 90 mm, the electrode length is 170 mm, and the sample amount is 20 mL. The charge of the physical characteristic parameters according to the applied voltage of the c-DBD plasma is shown in Figure 1d. The reactive species generated by c-DBD discharged into the liquid are shown in Figure 1e on optical emission spectroscopy. An N2 s positive band between 297 nm and 405 nm (C3Πu−B3Πg) was detected.

Plasma discharged in liquid produces various reactive species, including hydroxyl radicals, singlet oxygen, nitric oxide, and converted H_2_O_2_ as long-lived species. In particular, H_2_O_2_ and NOx are well-known factors related to cell proliferation and death. To compare ROS production in PTL created by c-DBD, we assessed H_2_O_2_ and NOx concentrations (Figure 1f,g). The concentration of H_2_O_2_ in the PTL of deionized (DI) water and culture medium with different plasma mixture ratios was assessed (Figure 1h). The H_2_O_2_ content gradually increased with increasing PTL ratio. The H_2_O_2_ content of the culture medium for PTL treatment was markedly higher than that of DI water. The results indicated that the change in the H_2_O_2_ content was affected by treatment for 20–40 min, after which point no significant increase was seen (data not shown).

### 3.2. Characterization of hUSLF

**Figure 2:**
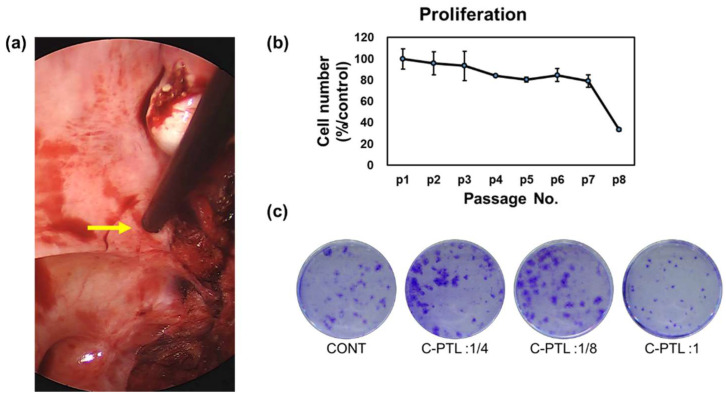
Characterization of hUSLF. Picture of uterosacral ligament during laparoscopic-assisted vaginal hysterectomy. The arrow indicates the uterosacral ligament (**a**). Proliferation of pelvic organ prolapse human uterosacral ligament fibroblasts (hUSLF) from passage 1 to passage 8 (**b**). Colony formation ability of hUSLF prepared with plasma-treated liquid in 1:4, 1:8, and 1:1 dilution (**c**).

The hUSLF were used to investigate the characterization of POP. The tissue was obtained via laparoscopic surgery. After the hUSLF were isolated from the uterosacral ligament tissue (Figure 2a, yellow arrow), cell proliferation was observed by passage to determine cell characteristics; cell numbers decreased rapidly after passage 7. The hUSLF were treated with the PTL and stained with crystal violet. The PLT treatment increased the number of cells. As shown in Figure 2c, the stained cells were grown in hUSLF after PTL treatment. The number of stained hUSLF increased compared to that of the control. Based on these results, we performed a series of PTL treatments that can be used for the clinical application of hUSLF to decrease the pathogenesis of POP.

### 3.3. Effect of PTL on Primary Cultured hUSLF

**Figure 3:**
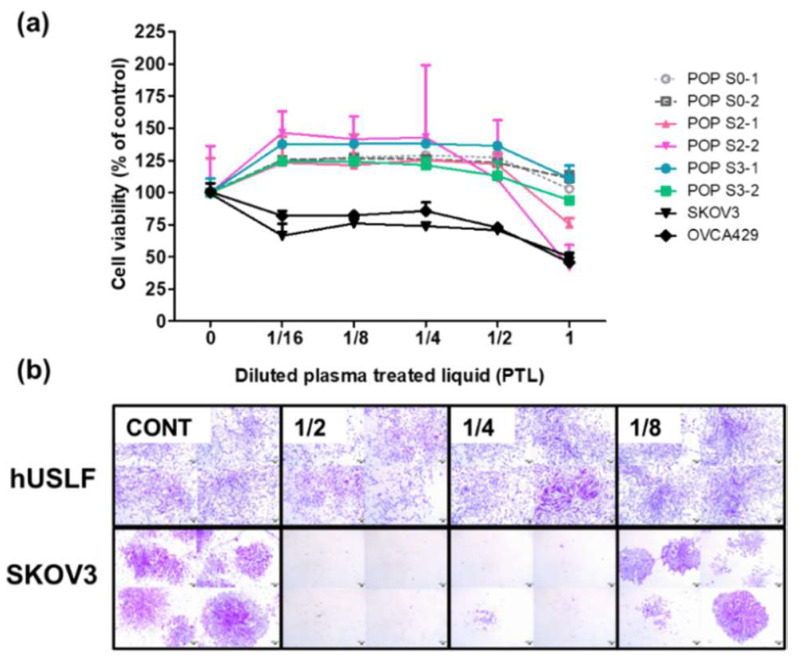
Cell viability of human uterosacral ligament fibroblasts (hUSLF) exposed to plasma-treated liquid (**a**). Cell viability was assessed under several conditions. A growth curve of the normal uterosacral ligament primary cells versus those of pelvic organ prolapse (POP) stage 0–1, stage 0–2, stage 2, stage 2–1, stage 2–2, stage 3, stage 3–1, and stage 3–2. SKOV3 and OVCA429 are ovarian cancer cell lines. (**b**) Colony-forming unit stain results are presented as proliferation changes. The differences between the crystal violet-stained cells were significant.

For the cell viability analysis, various dilutions of PTL were added to primary cultured hUSLF (stages 0, 2, and 3) and ovarian cancer cells (SKOV3 and OVCA429). Importantly, we found that cell viability was increased in all primary cultured hUSLF at all dilutions (Figure 3a; Table 2). Treatment with undiluted PTL did not significantly reduce hUSLF viability from POP stages 0 and 3, but a 25–59% decrease in cell survival was observed among hUSLF from POP stage 2. The ovarian cancer cell viability was inhibited under all PTL treatment conditions (Supplementary Figure S1). Consistent results were also found in the colony-forming analysis, in which the hUSLF primary cultured cells showed increased colony formation when treated with diluted PTL, while SKOV3 showed significantly decreased colony numbers, especially at the 1:2 and 1:4 dilutions (Figure 3b).

**Figure 4:**
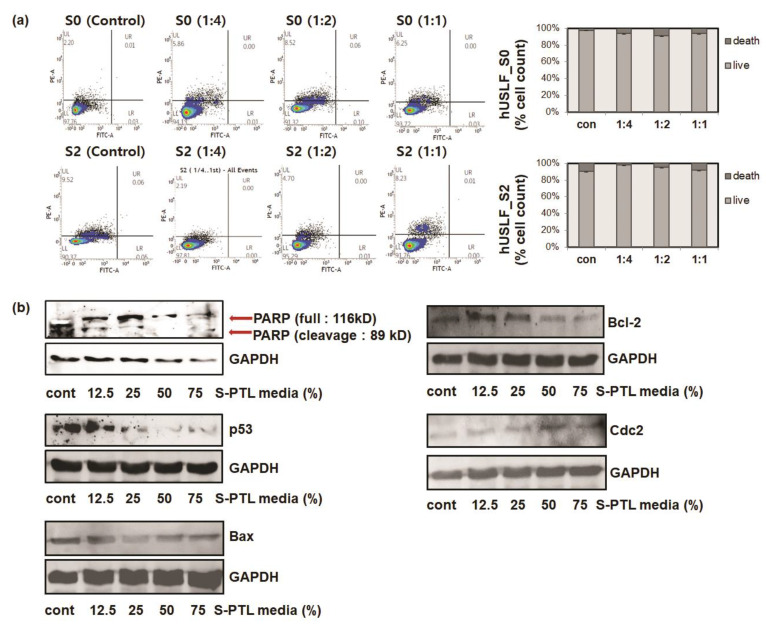
Apoptosis assay observed by Annexin V/propidium iodide (PI) staining and detected by flow cytometry (**a**). Recombinant Annexin V conjugated to fluorescein, the red-fluorescent PI nucleic acid binding dye. Platelet-treated liquid (PTL) treatment decreased human uterosacral ligament fibroblast (hUSLF) apoptosis at various dilution ratios. The hUSLF were exposed to 1:4 to 1:1 dilutions of PTL and cell culture medium. (**b**) The PARP and p53 expressions of hUSLF cells were inhibited by PTL treatment. Substrate cleavage by active PARP, Bax, Bcl-2, and Cdc2 was analyzed by immunoblotting with 1:8, 1:4, 1:2, and 1:1.3 ratios of PTL:cell culture medium.

Annexin V/propidium iodide staining was used to detect apoptosis (Figure 4a). The PTL induced apoptosis at rates of 25% (1:4 ratio) and 50% (1:2 ratio), rates that were markedly lower than that in the control group. Apoptotic cells were notably reduced in the PTL group exposed to low concentrations of PTL. The apoptosis rate did not change significantly in the normal-stage hUSLF after PTL treatment compared with the control and stage 2 cells.

To explore the possible mechanism by which PTL treatment affects hUSLF viability and regulates p53 protein expression, p53 related signaling pathways were investigated via Western blot. PTL treatment regulates the p53 signaling pathway and is transduced into intracellular signaling, including PARP, Bax, Bcl-2, and Cdc2. The results showed that pro-apoptotic proteins including RARP, p53, and Bax were downregulated by PTL treatment whereas anti-apoptotic Bcl-2 proteins and Cdc2 proteins were upregulated. In addition, expression levels associated with apoptosis signals, such as PARP and Bax, diminished when treated with PTL. In mammalian cells, p53 can induce not only cell death by apoptosis but also Cdks inhibitor p21 protein regulation. In apoptotic cells, tumor repressor p53 suppresses Bcl-2 and activates Bax. In addition, p53 can negatively regulate p21, which leads to downregulation of cyclin-dependent kinases such as CDK2 and Cdc2. The cyclin-dependent kinases are essential for cell cycle progression from G1 to S phase and G2 to M phase to control the cell cycle. Taken together, PARP, Bax, Bcl-2, and Cdc2 regulation by reduction in p53 expression through PTL treatment can promote the proliferation of hUSLF cells. These results imply that PTL may restore apoptotic cell death of hUSLF, which continuously undergo apoptosis.

## 4. Discussion

The treatment of POP depends on symptom severity. Treatment can include a variety of therapies such as (1) behavioral treatments, such as performing Kegel exercises designed to strengthen the pelvic floor muscles; (2) mechanical treatments, such as the insertion of a small plastic device called a pessary into the vagina to provide support for the drooping organs; and (3) surgical treatment to repair the affected tissue or organ or remove the organ (such as hysterectomy). Although POP is not a life-threatening disease, it significantly impacts quality of life. Based on our experimental results, if uterosacral ligament weakening is improved by plasma treatment (for which more research is needed), it may be a nonsurgical treatment option for patients with POP.

Plasma contains a variety of components, including electrons, ultraviolet photons, free radicals, charged ions, and neutral atoms. It has demonstrated a specific cellular mechanism by reactive oxygen and nitrogen species (RONS) produced by the discharge of non-thermal plasma. Ions and radicals generated by the plasma have high reactivity with other molecules and are in a gaseous state, so they can easily penetrate the surfaces of materials or narrow spaces into the cells, tissues, and organs. However, the gaseous state of plasma produces ozone and some ultraviolet rays that are unsuitable for use in a living environment or medication.

Indirect treatment is one way to convert non-thermal plasma into a medium by treating it in a liquid such as water or culture medium for cells and microorganisms. In our previous studies, we investigated plasma using the same intracellular cascade mechanism as direct and indirect treatment on normal cells, cancer cells, and bacteria [25,26,27]. It has the enormous advantage of being PTL, which is easier to use in the tissues and organs than existing plasma. The present study aimed to investigate the effects of PTL-induced RONS on cell viability and proliferation that suppress cellular apoptosis in POP patients after NBP treatment. In this study, we suggest that PTL treatment can attenuate the p53 signal pathway, which can increase the cell survival pathway and inhibit the apoptosis pathway.

Some evidence suggests that connective tissue abnormalities may contribute to POP [28]. Key elements of tissue stability are the quantity, ultrastructure, and organization of extracellular matrix proteins such as collagens, fibronectin, elastin, and their receptors such as integrins [29]. NBP reportedly stimulated collagen production both in vivo and in vitro [30,31].

Taken together, the NBP generated by c-DBD can lead to hUSLF proliferation of POP patient tissue and may be a clinical application of NBP as an innovative approach to treating POP.

## 5. Conclusions

Plasma medicine is an emerging field that comprises physics, biology, chemistry, engineering, and clinical medicine. The effects on anticancer, antibacterial, antiviral, anti-inflammatory, and wound healing were investigated. This is an especially useful application for cancer therapy, which can kill the cancer cells with few effects on normal cells. For plasma medicine, excited gas could dominate, but there are numerous particles similar to a cocktail mixture given the numerous effects of plasma such as disinfection, tooth whitening, and skin toning.

POP is associated with risk factors, such as parity, age, ethnicity, increased intra-abdominal pressure, menopause and estrogen deficiency, smoking, and neurological injury. There is some evidence that abnormalities of the connective tissue composition may contribute to the genesis of POP. However, POP treatment methods are very limited.

This study hypothesized that the application of low-dose non-thermal biocompatible plasma (NBP) to human disease (pelvic organ prolapse) would inhibit continuous apoptotic behaviors through blocking the p53 mechanism, which is the world’s first attempt. The NBP can induce colony formation, proliferation, and reduce apoptosis on these cells, which might lead to enhanced survival rate. In our former studies, they showed significantly high expression levels of the cell cycle arrest-related p21 gene, angiogenesis-related vascular endothelial growth factor (VEGF) gene, and p38 MAPK protein expression. Thus, c-NBP plasma could be used as a promising tool for an enhanced viable rate on hUSLF for the patient.

## Figures and Tables

**Table 1:** Clinical characteristics of women with and without uterine prolapse.

	POP-Group (n = 20)	No-POP-Group (n = 20)	*p* Value
AGE (years) (mean ± SD)	63.6 ± 10.4	66.1 ± 8.2	NS
BODY MASS INDEX (kg/m^2^) (mean ± SD)	25.4 ± 2.5	25.5 ± 2.3	NS
PARITY (median, range)	2.8 (2–4)	2.4 (1–4)	NS
MENOPAUSE (n, %)	17 (85)	19 (95)	NS

Abbreviation: POP, Pelvic organ prolapse. SD, standard deviation. NS, not significant.

**Table 2:** Cell viability (% of control).

Dilution Factor	POP S0–1	POP S0–2	POP S2–1	POP S2–2	POP S3–1	POP S3–2	SKOV3	OVCA429
0	100.0 ± 10.9	100.0 ± 1.4	100.0 ± 25.9	100.0 ± 36.4	100.0 ± 11.0	100 ± 2.5	100.0 ± 8.0	100.0 ± 1.2
1/16	124.4 ± 3.7	125.4 ± 0.7	123.0 ± 20.4	136.6 ± 16.8	137.7 ± 2.2	124.5 ± 2.6	66.5 ± 9.4	81.9 ± 4.0
1/8	127.4 ± 3.3	126.6 ± 1.2	121.3± 24.1	141.6 ± 17.6	137.9 ± 1.4	124.2 ± 3.6	76.0 ± 4.3	82.3 ± 1.9
1/4	129.0 ± 0.9	126.0 ± 2.6	125.3 ± 2.8	142.9 ± 56.3	138.1 ± 0.9	121.3 ± 5.2	74.0 ± 2.5	85.8 ± 6.7
1/2	127.2 ± 4.3	123.2 ± 2.8	122.0 ± 8.1	110.5 ± 45.7	136.5 ± 1.5	113.0 ± 11.1	70.7 ± 2.5	72.8 ± 1.8
1	102.9 ± 13.1	111.2 ± 5.6	75.4 ± 4.6	41.9 ± 17.2	111.1 ± 9.8	94.2 ± 3.1	50.3 ± 1.5	45.6 ± 3.7

## Data Availability

All data generated or analyzed during this study are included in this published article (and its Supplementary Materials).

## Supplementary Materials

The following are available online at https://www.mdpi.com/article/10.3390/cells10102728/s1, Figure S1: Cell viability treated with PTL according disease stage of pop and ovarian cancers compared with control which is stage 0 to stage 1. Table S1: Components of cell culture media. Appearance red transparent solution with pH 7.0 to 7.6 at room temperature. 314~347 mOsm/kg H_2_O of osmolality and less than or equal to 1.0 EU/ml of endotoxin.
