# Supplementary material for: Improvement of Cell Growth of Uterosacral Ligament Fibroblast Derived from Pelvic Organ Prolapse Patients by Cold Atmospheric Plasma Treated Liquid
Source: Cells. 2021 Oct 13;10(10):2728. doi: 10.3390/cells10102728 (PMC8534575; doi:10.3390/cells10102728)
Supplement: Supplementary file 1 [file cells-10-02728-s001.zip › cells-1387887-supplementary.pdf]

Supplementary Figure S1. Cell viability treated with PTL according disease stage of pop and ovarian cancers compared with control which is stage 0 to stage 1.

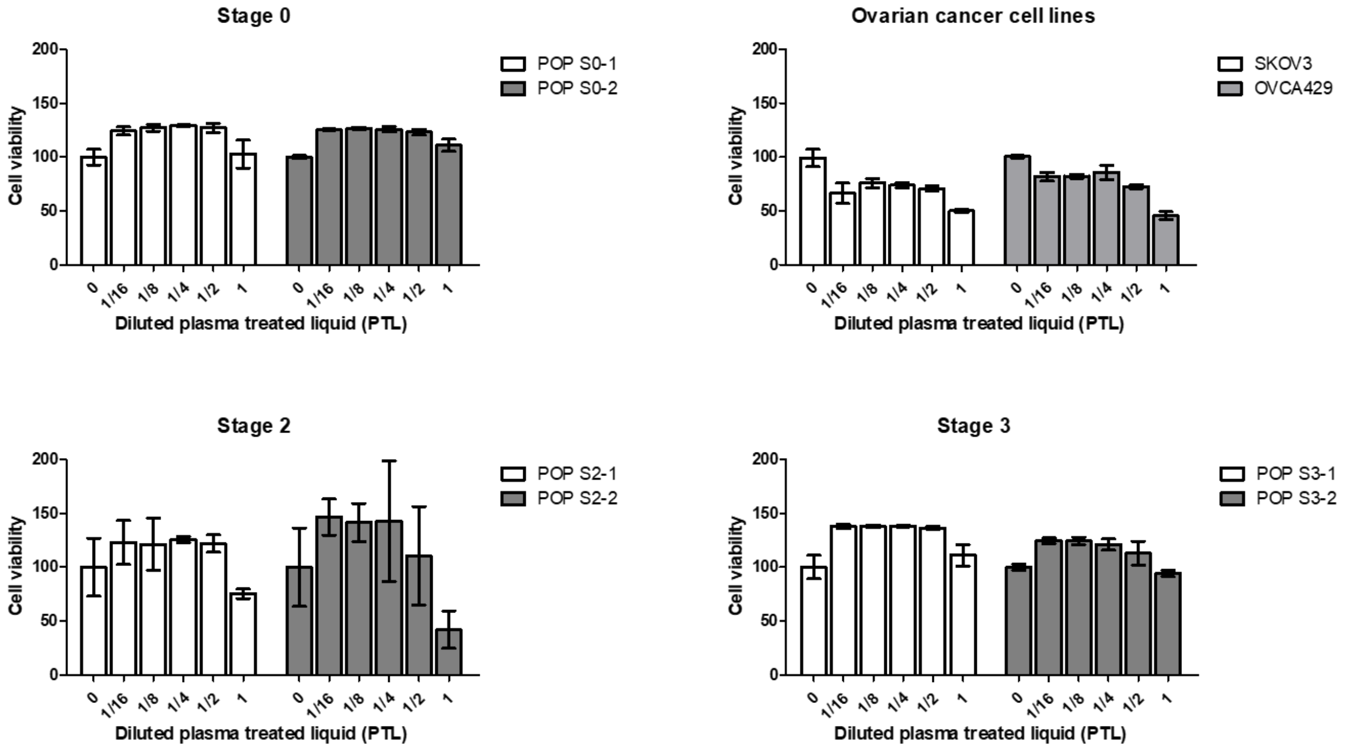

Supplementary Table S1. Components of cell culture media. Appearance red transparent solution with pH 7.0 to 7.6 at room temperature. 314~347 mOsm/kg H_2O of osmolality and less than or equal to 1.0 EU/ml of endotoxin.

components	mg/L
CaCl2 (anhydrous)	200.00
Fe(NO3)3·9H2O	0.10
KCl	400.00
MgSO4 (anhydrous)	97.67
NaCl	6400.00
NaHCO3	3700.00
NaH2PO4·H2O	125.00
D-Glucose	4500.00
Phenol Red	15.00
Sodium Pyruvate	110.00
L-Arginine·HCl	84.00
L-Cystine·2HCl	63.00
L-Glutamine	584.00
Glycine	30.00
L-Histidine·HCl·H2O	42.00
L-Isoleucine	105.00
L-Leucine	105.00
L-Lysine·HCl	146.00
L-Methionine	30.00
L-Phenylalanine	66.00
L-Serine	42.00
L-Threonine	95.00
L-Tryptophan	16.00
L-Tyrosine·2Na·2H2O	104.00
L-Valine	94.00
D-Ca Pantothenate	4.00
Choline Chloride	4.00
Folic Acid	4.00
i-Inositol	7.20
Niacinamide	4.00
Riboflavin	0.40
Thiamine·HCl	4.00
Pyridoxine·HCl	4.00
